# The contribution of *Alu* exons to the human proteome

**DOI:** 10.1186/s13059-016-0876-5

**Published:** 2016-01-28

**Authors:** Lan Lin, Peng Jiang, Juw Won Park, Jinkai Wang, Zhi-xiang Lu, Maggie P. Y. Lam, Peipei Ping, Yi Xing

**Affiliations:** Department of Microbiology, Immunology & Molecular Genetics, University of California, Los Angeles, Los Angeles, CA 90095 USA; Regenerative Biology, Morgridge Institute for Research, Madison, WI 53707 USA; Department of Computer Engineering and Computer Science, University of Louisville, Louisville, KY 40292 USA; KBRIN Bioinformatics Core, University of Louisville, Louisville, KY 40202 USA; Department of Physiology, University of California, Los Angeles, Los Angeles, CA 90095 USA; Department of Medicine, University of California, Los Angeles, Los Angeles, CA 90095 USA

**Keywords:** *Alu*, Transposable element, Exon, Alternative splicing, RNA editing, RNA-seq, Ribo-seq, Transcriptome, Proteome, Evolution, Primate

## Abstract

**Background:**

*Alu* elements are major contributors to lineage-specific new exons in primate and human genomes. Recent studies indicate that some *Alu* exons have high transcript inclusion levels or tissue-specific splicing profiles, and may play important regulatory roles in modulating mRNA degradation or translational efficiency. However, the contribution of *Alu* exons to the human proteome remains unclear and controversial. The prevailing view is that exons derived from young repetitive elements, such as *Alu* elements, are restricted to regulatory functions and have not had adequate evolutionary time to be incorporated into stable, functional proteins.

**Results:**

We adopt a proteotranscriptomics approach to systematically assess the contribution of *Alu* exons to the human proteome. Using RNA sequencing, ribosome profiling, and proteomics data from human tissues and cell lines, we provide evidence for the translational activities of *Alu* exons and the presence of *Alu* exon derived peptides in human proteins. These *Alu* exon peptides represent species-specific protein differences between primates and other mammals, and in certain instances between humans and closely related primates. In the case of the RNA editing enzyme ADARB1, which contains an *Alu* exon peptide in its catalytic domain, RNA sequencing analyses of A-to-I editing demonstrate that both the *Alu* exon skipping and inclusion isoforms encode active enzymes. The *Alu* exon derived peptide may fine tune the overall editing activity and, in limited cases, the site selectivity of ADARB1 protein products.

**Conclusions:**

Our data indicate that *Alu* elements have contributed to the acquisition of novel protein sequences during primate and human evolution.

**Electronic supplementary material:**

The online version of this article (doi:10.1186/s13059-016-0876-5) contains supplementary material, which is available to authorized users.

## Background

Transposable elements have played important roles in the evolution of gene regulation and function [1]. The primate-specific *Alu* retrotransposon is the most abundant class of transposable elements in the human genome, with over 1 million copies occupying over 10 % of the human genomic DNA [2]. Although *Alu* elements were historically considered as ‘junk DNA’, extensive research in the past two decades has revealed significant contributions of *Alu* to the evolution of the human genome and gene regulatory networks [1–3].

*Alu* is a major contributor to *de novo* origination of lineage-specific exons in primates [4]. Because the *Alu* element contains multiple sites that resemble the consensus donor and acceptor splice site signals, the insertion of *Alu* elements into intronic regions of existing genes produces preferable substrates for subsequent mutations that create and establish new exons [4, 5]. The exonization of *Alu* elements is frequent during primate and human evolution – thousands of human genes contain *Alu*-derived exon segments in their mRNA transcripts [6, 7]. Although the majority of *Alu* exons have low splicing activities and probably represent non-functional evolutionary intermediates, a subset of *Alu* exons have acquired ubiquitously strong or tissue-specific splicing activities in human tissues, as demonstrated by recent transcriptome studies using splicing-sensitive exon microarray and deep RNA sequencing [8, 9]. These ‘established’ *Alu* exons are preferentially located in the mRNA 5’ untranslated regions (5’-UTR) and may play a role in regulating mRNA translational efficiency [6, 8, 9]. *Alu* exons inserted into coding regions of protein-coding genes frequently contain premature termination codons (PTCs) and may provide a mechanism for fine-tuning steady-state mRNA levels by inducing mRNA nonsense-mediated decay (NMD) [10]. Together, these data have established the regulatory roles of *Alu* exons in multiple aspects of RNA metabolism including translation and degradation.

Despite the prevalence of *Alu* exons in the human transcriptome, the contribution of *Alu* exons to the human proteome remains unclear and controversial [11–15]. In the 1990s and early 2000s, Makałowski and others carried out large-scale discoveries of *Alu* exons in human genes using cDNA sequences and expressed sequence tags (ESTs) [11, 12, 16]. They identified numerous instances of ‘in-frame’ *Alu* exons in the coding region of human mRNAs that are predicted to add *Alu*-derived peptides to the protein products. However, the presence of an in-frame *Alu* exon in the mRNA coding sequence does not guarantee its translation and incorporation into stable protein products. Additionally, since most *Alu* exons have low splicing activities [6, 16], even a ‘translatable’ coding-region *Alu* exon may only be incorporated into a small fraction of the gene’s transcript products and consequently become largely undetectable and negligible on the protein level. In fact, in these studies there was little experimental evidence to support the existence of *Alu*-derived peptides *in vitro* or *in vivo*. In 2006, Makalowski and colleagues revisited this question by searching for transposable elements derived peptides in non-redundant protein entries in the Protein Databank (PDB) [13]. Since all proteins in PDB have solved 3D structures, Makalowski and colleagues reasoned that PDB provides a high-confidence collection of stable, functional proteins. They did not identify any *Alu* derived peptides in PDB protein entries. On the basis of this result, Makalowski and colleagues concluded that exons derived from *Alu* or other young repetitive elements do not have adequate evolutionary time to be incorporated into stable protein products, and the role of *Alu* exons should be almost entirely regulatory [13]. Since then, this has become the prevailing view on the contribution of *Alu* exons to the human proteome [3, 14, 15, 17]. However, this PDB-based study also has major drawbacks. Most importantly, PDB has very limited coverage of alternatively spliced protein isoforms, and less than 10 protein isoforms had structures deposited in PDB by the time of this study [18]. When structural biologists select proteins for structural characterization, there is an inherent bias towards selecting evolutionarily conserved protein isoforms and against protein isoforms with non-conserved (lineage-specific) exon segments such as *Alu* exons. Therefore, the presence of *Alu* exon peptides in the human proteome may be significantly underestimated in the PDB-based analysis. Indeed, in gene-specific studies, researchers have found evidence for the expression of *Alu* exon peptides *in vitro* or *in vivo* – two examples being the *Alu* exons in genes encoding the RNA editing enzyme ADARB1 [19] and DNA methyltransferase DNMT1 [20]. Neither case was recapitulated in the PDB study [13]. In summary, despite substantial interest in this topic, the real contribution of *Alu* exons to the human proteome remains unclear, and past studies may have reached dramatically different conclusions due to inherent limitations of their data sources.

In this work, we adopted a proteotranscriptomics approach to systematically assess the contribution of *Alu* exons to the human proteome. Using RNA sequencing (RNA-seq), ribosome profiling (Ribo-seq), and mass spectrometry data of diverse human tissues and cell lines, we evaluated the evidence for the translational activities of *Alu* exons and the presence of *Alu* exon derived peptides in human proteins.

## Results

### RNA-seq discovery of putative coding *Alu* exons with high splicing activities

RNA-seq has become a powerful approach for quantitative analyses of exon splicing [21]. We previously used an RNA-seq dataset of human cerebellum to characterize the splicing profiles of *Alu* exons [9]. Among *Alu* exons with high splicing levels (>50 % exon inclusion) in the cerebellum according to this RNA-seq analysis, the vast majority (over 80 %) were located in the mRNA 5’-UTR, and only a handful were located within the protein-coding region [9]. In the present work, to comprehensively identify putative coding *Alu* exons and characterize their splicing activities, we used a much larger RNA-seq dataset with approximately 1.8 billion RNA-seq reads covering 19 human tissues, including 16 tissues from the Illumina Human Body Map 2.0 dataset and three tissues from different anatomical compartments of the human placenta (see Materials and Methods).

**Fig. 1:**
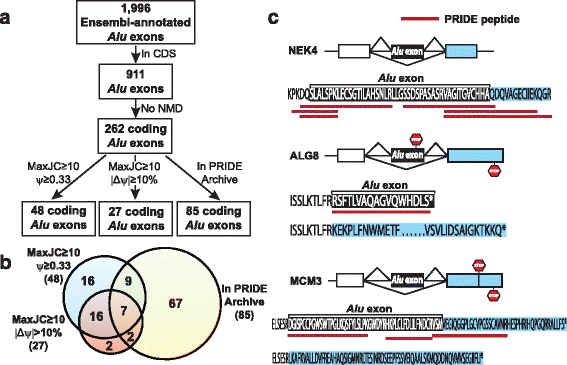
Identification and analysis of putative coding *Alu* exons in human genes. **a** The bioinformatics work flow to identify and characterize putative coding *Alu* exons using RNA-seq and proteomics data. **b** The Venn diagram illustrating the number of putative coding *Alu* exons with different types of RNA-seq or proteomic evidence. **c** The splicing patterns and deduced peptide sequences of putative coding *Alu* exons with supporting peptide sequences from the PRIDE database. The stop codon of each isoform is indicated by a STOP sign

We implemented a computational pipeline to identify putative coding *Alu* exons with high splicing activities (Fig. 1a and Materials and Methods). Briefly, from Ensembl transcript annotations, we extracted 1,996 *Alu*-derived internal (spliced) exons. Of these, 911 *Alu* exons were located within the coding sequences (CDS). To remove *Alu* exons that would trigger transcript degradation via mRNA nonsense-mediated decay (NMD), we further identified 262 coding-region *Alu* exons that would not introduce premature termination codons (PTCs) within or downstream of the *Alu* exons when included into the transcripts. Specifically, PTCs were defined as stop codons located more than 50 bp upstream of the last exon-exon junction of the mRNA [22]. These 262 non-PTC exons were considered as candidate protein-coding *Alu* exons that had the potential to add *Alu*-derived peptides to the protein products. Of note, 69 % (182/262) of these candidate protein-coding *Alu* exons were divisible by 3 in exon length while only 24 % (159/649) of the putative NMD-inducing *Alu* exons were divisible by 3 (Fisher’s exact test *P* = 2e-36). Next, we performed detailed RNA-seq analyses on these exons to identify those with moderate-to-high levels of splicing or differential splicing activities among human tissues. For each exon, we calculated its transcript inclusion level (denoted as percent-spliced-in, or Ψ [23]) in a given tissue using RNA-seq reads uniquely mapped to the upstream, downstream, and exon-skipping splice junctions (see Materials and Methods). To ensure accurate estimation of the transcript inclusion level, we required that at least one splice junction (upstream, downstream, or exon-skipping) had at least 10 reads. We identified 48 *Alu* exons with transcript inclusion levels of at least 33 % in one of the human tissues, indicating moderate-to-high levels of exon splicing. In parallel, we performed pairwise tissue comparisons using the MATS algorithm for differential splicing analysis [24] and identified 27 *Alu* exons with at least 10 % change in transcript inclusion levels (that is, |ΔΨ| ≥10 %) between at least one pair of tissues (false discovery rate (FDR) <10 %), indicating differential splicing among human tissues. The vast majority of these differentially spliced *Alu* exons (23/27) were also among the 48 exons with at least 33 % transcript inclusion levels in at least one of the human tissues. In total, we obtained 52 *Alu* exons by combining these two exon lists (Fig. 1b and Additional file 1: Table S1). These results indicate that although *Alu* exons with high splicing levels are preferentially located within the 5’-UTR [6, 8, 9], there is a reasonably large number of putative protein-coding *Alu* exons with high splicing activities in human tissues.

### Translational and proteomic evidence for protein-coding *Alu* exons

To systematically assess the proteomic evidence for *Alu* exon derived peptides in human proteins, we queried the 262 putative coding *Alu* exons in Ensembl transcripts (Fig. 1a) against the PRoteomics IDEntifications Database (PRIDE) [25]. PRIDE is a comprehensive repository for proteomics data, containing protein and peptide sequences as well as their supporting spectral evidence from mass spectrometry experiments. We searched PRIDE peptide sequences against the putative open reading frames (ORFs) of *Alu* exon inclusion transcripts as well as the ORFs of all other human transcripts. We excluded peptide sequences mapped to multiple locations in human ORFs. Then we defined a putative protein-coding *Alu* exon as having a peptide match in PRIDE if there was a peptide sequence uniquely mapped to the translated peptide sequence from the exon body or the splice junctions spanning the *Alu* exon and its upstream or downstream flanking exon. Of the 262 putative coding *Alu* exons, 85 had peptide evidence in the PRIDE database (Fig. 1a and Additional file 2: Table S2). Of note, 69 of these 85 (81 %) *Alu* exons had peptide sequences crossing the upstream or downstream splice junctions (Additional file 2: Table S2). Because the flanking exons of most *Alu* exons are not derived from repetitive elements, these splice junction spanning peptide sequences provide stronger evidence that these putative coding *Alu* exons are translated into protein sequences. Among the 52 putative protein-coding *Alu* exons with RNA-seq evidence for high splicing activities in human tissues, 18 had peptide matches in PRIDE (Fig. 1b and Table 1). A few examples are shown in Fig. 1c. In NEK4 (ENSG00000114904) we found six peptide sequences in PRIDE that mapped to the upstream or downstream splice junction of the *Alu* exon. In ALG8 (ENSG00000159063), the *Alu* exon was predicted to encode a different C-terminal peptide of the protein – this *Alu* exon peptide was supported by a peptide sequence in PRIDE. Another example was MCM3 (ENSG00000112118). In this gene, *Alu* exon inclusion caused a frameshift in the downstream 3’ terminal exon, which resulted in a distinct C-terminal peptide by using a different reading frame within the same terminal exon. We found peptide sequences in PRIDE that mapped to either the *Alu* exon or the downstream splice junction. In addition to the PRIDE analysis, we also manually searched the 52 putative protein-coding *Alu* exons with high splicing activities against a second mass spectrometry database PeptideAtlas [26]. Different from PRIDE, PeptideAtlas reprocessed the original protein mass spectrometry data using stringent FDR criteria [26]. Therefore, PeptideAtlas has a very low false positive rate at the expense of a higher false negative rate. Our manual search against PeptideAtlas identified the peptide evidence for *Alu* exons in SUGT1 (ENSG00000165416) and DNMT1 (ENSG00000130816), both were also supported by PRIDE (Table 1). Overall, among the 52 putative coding *Alu* exons with high splicing activities, 18 (35 %) had peptide evidence in either PRIDE alone or both PRIDE and PeptideAtlas. It should be noted that this percentage is expected to be an underestimate, since peptide identification from proteomics experiments is biased towards highly expressed proteins [27].

**Table 1:** Putative coding *Alu* exons with significant splicing activities and supporting peptide evidence in proteomics databases

	Gene symbol	Gene ID	Exon coordinate (hg19)	Gene description	MaxJC ≥10 ψ ≥0.33	MaxJC ≥10 |Δψ| ≥10 %	In PRIDE	In PeptideAtlas
1	SUGT1	ENSG00000165416	chr13:53235609-53235705	SGT1, suppressor of G2 allele of SKP1 (S. cerevisiae)	Yes	Yes	Yes	Yes
2	DNMT1	ENSG00000130816	chr19:10290862-10290910	DNA (cytosine-5-)-methyltransferase 1	Yes	Yes	Yes	Yes
3	C21orf7	ENSG00000156265	chr21:30463988-30464082	Chromosome 21 open reading frame 7	Yes	No	Yes	No
4	ADARB1	ENSG00000197381	chr21:46604388-46604508	Adenosine deaminase, RNA-specific, B1	Yes	Yes	Yes	No
5	ALG8	ENSG00000159063	chr11:77813919-77813994	Asparagine-linked glycosylation 8, alpha-1,3-glucosyltransferase homolog (S. cerevisiae)	Yes	No	Yes	No
6	NEK4	ENSG00000114904	chr3:52783707-52783845	NIMA (never in mitosis gene a)-related kinase 4	Yes	No	Yes	No
7	ZNF415	ENSG00000170954	chr19:53618462-53618560	Zinc finger protein 415	Yes	Yes	Yes	No
8	TPRKB	ENSG00000144034	chr2:73959710-73959827	TP53RK binding protein	Yes	No	Yes	No
9	C21orf7	ENSG00000156265	chr21:30463821-30463897	Chromosome 21 open reading frame 7	Yes	No	Yes	No
10	C11orf80	ENSG00000173715	chr11:66523823-66523976	Chromosome 11 open reading frame 80	Yes	No	Yes	No
11	ZNF573	ENSG00000189144	chr19:38249115-38249236	Zinc finger protein 573	No	Yes	Yes	No
12	MCM3	ENSG00000112118	chr6:52130032-52130183	Minichromosome maintenance complex component 3	No	Yes	Yes	No
13	SLC3A2	ENSG00000168003	chr11:62639048-62639141	Solute carrier family 3 (activators of dibasic and neutral amino acid transport), member 2	Yes	No	Yes	No
14	EIF4E	ENSG00000151247	chr4:99807604-99807697	Eukaryotic translation initiation factor 4E	Yes	Yes	Yes	No
15	KCNRG	ENSG00000198553	chr13:50592958-50593056	Potassium channel regulator	Yes	No	Yes	No
16	FAM124B	ENSG00000124019	chr2:225265097-225265222	Family with sequence similarity 124B	Yes	Yes	Yes	No
17	B3GALNT2	ENSG00000162885	chr1:235659495-235659618	Beta-1,3-N-acetylgalactosaminyltransferase 2	Yes	No	Yes	No
18	ZNF195	ENSG00000005801	chr11:3382972-3383119	Zinc finger protein 195	Yes	Yes	Yes	No

**Fig. 2:**
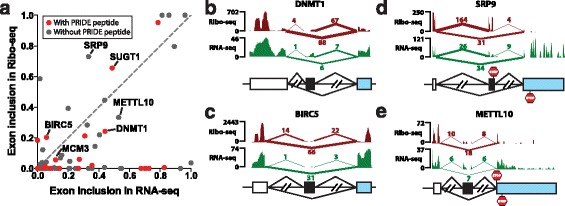
Ribo-seq data (HeLa cells) provide evidence for the translational activities of putative coding *Alu* exons. **a** Comparison of *Alu* exon inclusion levels in Ribo-seq and RNA-seq data of HeLa cells. Each dot represents an *Alu* exon. Seventy-six putative coding *Alu* exons with at least 10 reads mapped to one of the three splice junctions in both Ribo-seq and RNA-seq data are shown in the plot. Exons with PRIDE peptide sequences are indicated in red. **b**-**e** The UCSC genome browser view of the Ribo-seq and RNA-seq data of four representative *Alu* exons. The upstream junction read count (UJC), downstream junction read count (DJC), and skipping junction read count (SJC) are also indicated. The stop codon of each mRNA isoform is indicated by a STOP sign

To obtain an independent line of evidence for the translation of these putative coding *Alu* exons into protein products, we analyzed the footprint of ribosomes on *Alu* exons using ribosome profiling (Ribo-seq) data. Ribo-seq has recently been developed as a powerful approach for high-throughput analyses of protein translation [28]. Here we jointly analyzed the Ribo-seq and RNA-seq data of HeLa cells [29]. Specifically, we mapped the Ribo-seq and RNA-seq reads to the upstream, downstream, and exon-skipping splice junctions of *Alu* exons, and computed each *Alu* exon’s transcript inclusion levels in the Ribo-seq and RNA-seq data, respectively (Materials and Methods). To ensure reliable estimation of transcript inclusion levels, we restricted this analysis to *Alu* exons with at least 10 reads mapped to one of three splice junctions in both Ribo-seq and RNA-seq data. As shown in Fig. 2a, among the 76 putative coding *Alu* exons that met this criterion, 38 exons had non-zero transcript inclusion levels in the Ribo-seq data, indicating ribosome footprints translating through the *Alu* exons. Seventeen exons had transcript inclusion levels of at least 15 % in the Ribo-seq data. Of note, 15 *Alu* exons had higher transcript inclusion levels in the Ribo-seq data than in the RNA-seq data (Fig. 2a and Additional file 3: Table S3), suggesting that the *Alu* exon inclusion mRNA isoform was translated at a comparable or even higher rate as compared to the ancestral mRNA isoform lacking the *Alu* exon. The Ribo-seq and RNA-seq signals of four *Alu* exons were shown in Fig. 2b-e. These include *Alu* exons with PRIDE or PeptideAtlas evidence (DNMT1 (ENSG00000130816), BIRC5 (ENSG00000089685)), as well as those without (SRP9 (ENSG00000143742), METTL10 (ENSG00000203791)). Of note, in both SRP9 and METTL10, the *Alu* exon was the penultimate exon, and the stop codon of the *Alu* exon inclusion mRNA isoform was located either within the *Alu* exon (SRP9) or in the immediate downstream terminal exon (METTL10). In both cases the Ribo-seq signal diminished beyond the stop codon (Fig. 2d, e). We note that nine *Alu* exons with PRIDE peptide sequences had no Ribo-seq reads mapped to the exon inclusion splice junctions, including three exons with RNA-seq transcript inclusion levels of over 50 % (Additional file 3: Table S3). This may be attributed to cell-type-specific differences in protein translation between the HeLa cells and other tissues and cell types.

We found that putative protein-coding *Alu* exons had a lower percentage of exons derived from the AluY subfamily, which was the youngest subfamily of *Alu* elements in the human genome [30]. In total, 4.2 % (11/262) putative protein-coding *Alu* exons were AluY derived, compared to 7.0 % (121/1734) for other *Alu* exons. Moreover, protein-coding *Alu* exons with PRIDE evidence had the lowest percentage of AluY (2.4 %, 2/85). These differences were not statistically significant (Fisher’s exact test, *P* = approximately 0.1), which may be attributed to small sample size. However, the trend was interesting and consistent with the notion that it required time for *Alu* exons to strengthen splicing signals and develop protein-coding capabilities.

### Assessment of transcriptomic and proteomic evidence for protein-coding *Alu* exons

We performed a series of analyses to further assess the transcriptomic and proteomic evidence for protein-coding *Alu* exons. First, to validate the splicing of these exons, we randomly selected 20 protein-coding *Alu* exons, and used fluorescently labeled RT-PCR to verify their exon inclusion patterns and quantify their splicing levels in a diverse panel of 21 human tissues (Additional file 4: Table S4). Nineteen out of the 20 exons were validated to be spliced into the human transcriptome (Additional file 5: Figure S1). This high validation rate (19 out of 20) was consistent with our previous RT-PCR and sequencing confirmation of UTR *Alu* exons identified by RNA-seq [9], confirming that our RNA-seq identification of *Alu* exon splicing had a very high accuracy. Second, we further assessed the Ribo-seq signal of protein-coding *Alu* exons in HeLa cells, using putative NMD-inducing *Alu* exons in protein-coding genes as the control. Seventeen out of 76 protein-coding *Alu* exons with sufficient Ribo-seq coverage had transcript inclusion levels of at least 15 % in the Ribo-seq data, as compared to 24 out of 180 for putative NMD-inducing *Alu* exons. We observed an increase in Ribo-seq signals of protein-coding *Alu* exons (22 % vs. 13 %), although the statistical significance was marginal (*P* = 0.056, one-sided Fisher’s exact test), probably due to the small sample size and possibly the effect of the pioneering round of translation in NMD transcripts [31]. These data suggest that Ribo-seq provides a discriminative feature of protein-coding *Alu* exons but cannot be used alone as their sole evidence.

As the most central and direct evidence for protein-coding *Alu* exons in this work came from the analysis of PRIDE peptide data, we used multiple negative controls to assess the reliability of our PRIDE search. First, we used mouse peptides as a negative control and searched mouse peptides in PRIDE and in our own COPaKB database of cardiac proteins [32] against human *Alu* exons. We did not find any mouse peptide hit of longer than four amino acids in either database, which was below the length cutoff we required for human PRIDE peptide hits (≥6 amino acids). Second, we used putative NMD-inducing *Alu* exons in protein-coding genes as another negative control to assess the FDR of our PRIDE analysis using human peptide data. Specifically, we performed PRIDE search on all NMD-inducing *Alu* exons and identified PRIDE hits for 47 out of 649 exons (7.2 %). This percentage is significantly lower than that of the putative protein-coding *Alu* exons (32.4 %, 85 out of 262; *P* = 1.8e-20, Fisher’s exact test). Even if we take a very conservative estimate that all of these 649 NMD-inducing exons represent true negatives and none of them can be protein-coding via mRNA isoforms with alternative reading frames or A-to-I editing of the STOP codon (a possible scenario supported by the literature; see [33]), we would estimate that 19 of the 262 putative protein-coding *Alu* exons would generate false positive PRIDE hits (that is, 262 × 7.2 %), yielding a low upper-bound estimate of FDR of 22 % (that is, 19 out of 85). In summary, using putative NMD-inducing *Alu* exons as our negative control, we demonstrate that the FDR of our PRIDE search is low, and that the vast majority of the 85 PRIDE hits reflect *bona fide* proteomic evidence for protein-coding *Alu* exons.

### Human-specific increase in the splicing activity of a protein-coding *Alu* exon in SUGT1

**Fig. 3:**
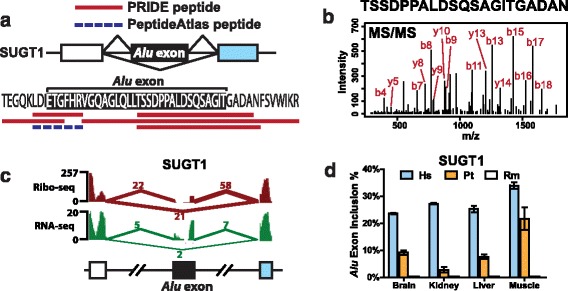
A protein-coding *Alu* exon in SUGT1 supported by multiple lines of proteomics and Ribo-seq evidence. **a** The splicing pattern and deduced peptide sequence of a putative coding *Alu* exon in SUGT1 and its corresponding peptide evidence from PRIDE and PeptideAtlas. **b** Tandem mass spectrometry (MS/MS) spectrum of the peptide TSSDPPALDSQSAGITGADAN from PRIDE (experiment ID: 26855, spectrum ID: 7275). **c** The UCSC genome browser view of the Ribo-seq and RNA-seq data of the SUGT1 *Alu* exon. **d** RT-PCR analysis of the SUGT1 *Alu* exon in four different tissues in human (Hs), chimpanzee (Pt), and rhesus macaque (Rm). Error bars show standard error of the mean from at least three replicate experiments

We studied an *Alu* exon derived peptide in SUGT1, which was supported by multiple lines of evidence from proteomics (PRIDE and PeptideAtlas) and Ribo-seq data (Fig. 3a-c). SUGT1 is an assembly factor for the kinetochore and is required for the G1/S and G2/M transitions [34]. The *Alu* exon encodes a peptide of 33 amino acids inserted at the end of the third tetratricopeptide repeat (TPR) in the SUGT1 protein product. We identified five peptide sequences in PRIDE that matched to the upstream or downstream splice junction of this *Alu* exon, and additional peptide evidence was found in the highly stringent PeptideAtlas (Fig. 3a). This exon had a significant splicing activity in HeLa cells with a transcript inclusion level of 49 % according to RNA-seq data, while its transcript inclusion level in the Ribo-seq data was even higher (66 %; see Figs. 2a and 3c), suggesting active translation of the *Alu* exon inclusion mRNA isoform. Given the strong evidence for this *Alu* exon derived peptide, we next asked whether the expression of this *Alu* exon inclusion protein isoform has been detected in previous studies. A literature search showed that the SUGT1 protein isoform containing the 33 amino acids encoded by exon 6 (that is, the *Alu* exon) was first identified as a doublet band in Hela cell extracts, as well as in human tissues including brain, liver, lung, and testis, and termed as SUGT1B, while the exon 6 skipping protein isoform was called SUGT1A [35]. The authors thought SUGT1B (that is, the exon inclusion isoform) was the ancestral full-length isoform while SUGT1A (that is, the exon skipping isoform) represented an alternative isoform of the gene. Later studies also detected SUGT1B protein in HeLa, THP-1, and 293T cell extracts [34, 36]. However, it was not recognized that exon 6 was derived from a primate specific *Alu* retrotransposon [34–36].

The recent origin of *Alu* elements suggests that the *Alu* exon derived peptide in SUGT1 could contribute to the evolutionary divergence of the human and non-human primate protein products. To investigate the evolution of this *Alu* exon further, we designed RT-PCR primers for its flanking constitutive exons in human, chimpanzee, and rhesus macaque genomes and quantified the exon splicing levels in four tissues (brain, kidney, liver, and muscle) (Fig. 3d; also see Additional file 5: Figure S2 for the fluorescently labeled RT-PCR gel images). Our RT-PCR analyses showed this exon had the highest levels of splicing (12–35 %) in human tissues. By contrast, the orthologous exon region was completely absent from the rhesus macaque transcripts. Chimpanzee transcripts showed intermediate transcript inclusion levels (0–24 %).

Next we aligned the human, chimpanzee, and rhesus genomic sequences with the consensus sequence of the corresponding *Alu* subfamily (*AluSx*) (Additional file 5: Figure S3). The resulting alignment suggested that this *Alu* exonization event occurred prior to the most recent common ancestor of humans and chimpanzees. Specifically, a C to T substitution created the 5’ splice site GT dinucleotide, which, when combined with the pre-existing 3’ splice site AG dinucleotide in *AluSx*, led to *Alu* exonization. Additionally, a G to A substitution at the +3 intronic position of the 5’ splice site strengthened the splice site score from 5.29 to 9.46 as calculated by MAXENT [37], representing an over 16-fold increase in the likelihood of matching to the consensus MAXENT 5’ splice site model. The rhesus sequence lacked the essential GT dinucleotide at the 5’ splice site, consistent with the observation that the exon was completely skipped in rhesus transcripts. We did not observe any nucleotide difference between the human and chimpanzee sequences within the exon or the 5’ and 3’ splice site regions (Additional file 5: Figure S3). However, beyond the splice sites there were a number of nucleotide differences in the upstream and the downstream intronic regions, which may be responsible for the difference in splicing levels between the human and chimpanzee exons.

**Fig. 4:**
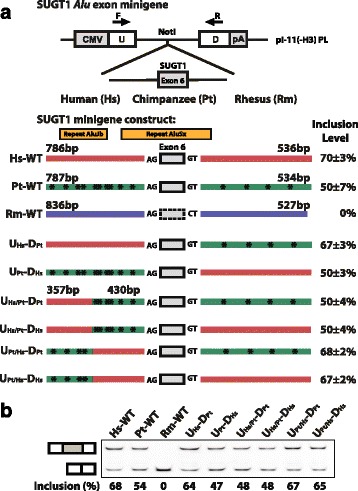
Minigene splicing reporter analysis of the human-specific splicing change of SUGT1 protein-coding *Alu* exon (Exon 6). **a** Schematic diagrams of the pI-11-H3 minigene splicing reporter and the wild-type/mutant minigene constructs of SUGT1 *Alu* exon. The nucleotide differences between human and chimpanzee are indicated by asterisks on the chimpanzee sequence. **b** Representative gel image for fluorescently labeled RT-PCR analyses in Hela cells using the SUGT1 *Alu* exon wild-type and mutant minigene constructs

We carried out a comprehensive investigation of the genomic sequence changes that strengthened the SUGT1 *Alu* exon in the human lineage (Fig. 4). There was no obvious candidate for causal *cis* sequence change(s) based on sequence analysis of splicing regulatory elements. Between human and chimpanzee, no sequence divergence was found within the *Alu* exon or in the 9 bp 5’ splice site and 23 bp 3’ splice site regions. Therefore, the causal *cis* sequence change(s) must reside in the upstream or downstream intronic region further away from the splice sites. These intronic regions contained a fairly large number of sequence changes between human and chimpanzee. We cloned a large genomic fragment surrounding the SUGT1 *Alu* exon into a minigene splicing reporter (see Materials and Methods) and generated three splicing reporter constructs corresponding to the wild-type human, chimpanzee, and rhesus genomic sequence (Hs-WT, Pt-WT, and Rm-WT; see Fig. 4a). When expressed in Hela cells, the chimpanzee minigene construct had an exon inclusion level of 50 ± 7 %, while the human minigene construct had a higher exon inclusion level of 70 ± 3 %, and no exon inclusion was observed for the rhesus minigene construct. The splicing difference between the human and chimpanzee wild-type minigene constructs was consistent with the difference of endogenous splicing levels in human and chimpanzee tissues (12–35 % vs. 0–24 %). The overall higher baseline exon inclusion levels in the minigene constructs may indicate deeper intronic splicing silencers that were not cloned into the minigene reporter, but this should not affect our human vs chimpanzee comparative analysis. Then we used a sequence swapping strategy [38] to make six additional splicing reporter constructs in which genomic segments from different species were swapped in order to narrow down the genomic region responsible for the human-specific splicing pattern (Fig. 4a). The analysis of these minigene splicing reporters indicates that a proximal 430 bp upstream intronic region is responsible for the human-specific increase in splicing compared to chimpanzee. All minigene constructs containing the chimpanzee version of this 430 bp intronic region showed approximately 50 % exon inclusion, while all minigene constructs containing the human version of this region showed close to 70 % exon inclusion, despite being placed within different surrounding sequence context (Fig. 4a, b). We obtained similar results when transfecting these reporters to a chimpanzee fibroblast cell line (data not shown), further supporting our hypothesis that the splicing divergence of this SUGT1 *Alu* exon was driven by *cis* sequence changes. Within this 430 bp upstream intronic region, there were six individual nucleotide substitutions between human and chimpanzee. We were unable to successfully perform further point mutation analyses of this region, due to the sequence homology between this region (which was AluSx derived) with an adjacent upstream AluJb element (see Fig. 4a). Nonetheless, our minigene splicing reporter data have established the role of specific *cis* genomic sequences in shaping the evolution of this *Alu* exon in the human lineage.

Collectively, our data indicate a gradually strengthened splicing activity of the SUGT1 *Alu* exon during recent primate and human evolution. It is possible that the acquisition and increased expression of this *Alu*-derived peptide in human tissues have certain adaptive benefits and are driven by positive selection.

### The *Alu* exon inclusion isoform of ADARB1 encodes an active RNA editing enzyme with an altered editing activity

**Fig. 5:**
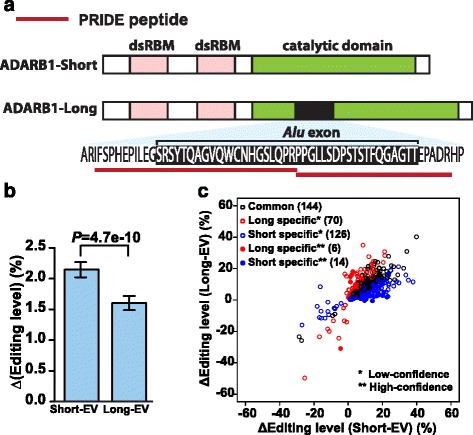
ADARB1 *Alu* exon inclusion isoform encodes an active RNA editing enzyme with altered editing activity. **a** The schematic diagram of the protein domain structure of ADARB1 isoforms and supporting peptide sequences from the PRIDE database. **b** The change in overall RNA editing levels of 7,618 RNA editing sites in HEK293 cells upon ectopic expression of the exon skipping (Short) or the exon inclusion (Long) ADARB1 isoform as compared to the empty vector (EV) control. Error bars show standard errors calculated based on the 7,618 known RNA editing sites used in this analysis. **c** Common and isoform-specific differentially edited sites upon ectopic expression of the *Alu* exon inclusion (Long) or skipping (Short) ADARB1 isoform. Isoform-specific differentially edited sites are further classified (low-confidence, high-confidence) based on the strength of the RNA-seq evidence

To investigate whether *Alu*-derived peptides can be part of functional proteins, we selected a protein coding *Alu* exon in the RNA editing enzyme ADARB1 (ENSG00000197381, also known as ADAR2) for detailed studies. The crystal structure of the catalytic domain of human ADARB1 has been solved [39]. The *Alu* exon encodes a 40 amino acid peptide inserted into the catalytic deaminase domain, which is supported by two peptide sequences in PRIDE (Fig. 5a). This exon displayed moderate to high levels of splicing (25–100 %) across human tissues according to the RNA-seq data, including eight out of 19 tissues with over 75 % exon inclusion levels. Our RT-PCR analyses of human, chimpanzee, and rhesus tissues showed that the *Alu* exon was consistently spliced into transcripts with comparable splicing levels in all three species, while there was variation in its splicing levels across different tissues and individuals (Additional file 5: Figure S4). In previous *in vitro* studies where purified recombinant ADARB1 protein isoforms were incubated with an artificial dsRNA substrate, the *Alu* exon inclusion protein isoform showed a lower catalytic activity than the *Alu* exon skipping isoform [19, 40]. However, the functional activity of the *Alu* exon inclusion ADARB1 protein isoform on endogenous mRNA transcripts has not been examined on a genome-scale in a live cell setting.

To test the effect of the ADARB1 protein isoforms on RNA editing, we selected the HEK293 cell line which had a low endogenous ADARB1 level, and ectopically expressed either the *Alu* exon inclusion or skipping ADARB1 protein isoform. Real-time qRT-PCR and western blot analyses indicated that both isoforms were expressed at similar levels (Additional file 5: Figure S5). To characterize the transcriptome-wide effect on RNA editing, we then performed strand-specific RNA-seq of cells transfected with one of the two ADARB1 protein isoforms or an empty vector control (Materials and Methods). We collected annotated A-to-I editing sites in human genes from the RADAR database of RNA editing sites [41], and restricted our analysis to a set of 7,618 sites with sufficient sequencing coverage in our data set (Materials and Methods). We then compared the overall RNA editing levels in the cells across different experimental conditions. Both the *Alu* exon inclusion and skipping protein isoforms significantly enhanced the overall editing levels in the HEK293 cells as compared to the empty vector control (Wilcoxon test, Short vs. EV: *P* = 6.5e-68, Long vs. EV: *P* = 2.1e-50; Fig. 5b), indicating the *Alu* exon inclusion protein isoform is an active RNA editing enzyme with a global impact on RNA editing. We also noted that the overall RNA editing level was lower in cells expressing the *Alu* exon inclusion isoform as compared to cells expressing the *Alu* exon skipping isoform (Wilcoxon test, *P* = 4.7e-10; Fig. 5b), consistent with the *in vitro* assay results on the synthetic RNA editing substrate [19, 40].

To investigate potential differences in the site selectivity of these two ADARB1 isoforms, we sought to identify differentially edited sites upon ectopic expression of the *Alu* exon inclusion (long) or skipping (short) isoform. Specifically, for each annotated RNA editing site in the RADAR database [41], we counted and compared their edited and unedited RNA-seq reads with those of the empty vector (EV) transfected cells. For each individual replicate, we used Fisher’s exact test to calculate the *P* value, then combined the *P* values of three replicates using Fisher’s method to generate a combined *P* value for differential editing, followed by correction of multiple testing to generate FDR. The editing sites with FDR ≤ 10 % were called differential RNA editing sites. We then compared the identified differential RNA editing sites for the *Alu* exon inclusion (long) or skipping (short) ADARB1 isoform to identify common and isoform-specific differential RNA editing sites. To guard against potential spurious calls of isoform-specific differential editing sites due to RNA-seq noise and statistical fluctuation, we also generated a high-confidence list of isoform-specific differential RNA editing sites, defined as those with FDR ≤ 10 % for one isoform and FDR ≥ 90 % for the other isoform. We identified a total of 360 differential RNA editing sites in the RNA-seq data after ectopic expression of one of the two isoforms (Fig. 5c). As seen in Fig. 5c, most of them were either common to the two isoforms, or were classified as isoform-specific but the difference between the two isoforms was minor and could be attributed to random RNA-seq noise and statistical fluctuation (that is, those classified as ‘low-confidence’ isoform specific sites). We did identify six high-confidence long isoform specific and 14 high-confidence short isoform specific differential RNA editing sites, but they represented a fairly small percentage of all identified differential RNA editing sites (Fig. 5c). It is interesting to note that in general, we identified almost twice as many short isoform specific sites than long isoform specific sites, at various confidence level cutoffs (Fig. 5c). Collectively, these data suggest that regulated alternative splicing of this *Alu* exon may fine tune the editing activities and in limited cases the target site selectivity of the ADARB1 protein products.

## Discussion

The creation and establishment of new exons provide an important evolutionary strategy for generating genetic novelties in existing genes [42, 43]. A large body of work has investigated the exonization of *Alu* elements as a major source for new exons during primate and human evolution [5, 7–9, 44–47]. Recent studies have shown that a subset of *Alu* exons in human genes have acquired strong splicing activities [8, 9], and that they play a variety of regulatory roles at the RNA level such as the control of mRNA degradation and translation [8–10]. On the other hand, the contribution of *Alu* exonization to the human proteome has been considered to be minimal [13]. In 2006, a survey of protein sequence entries in the PDB did not identify any peptide segment derived from *Alu* or other young transposable elements (TEs), leading the authors to conclude that ‘functional proteins are unlikely to contain TE cassettes derived from young TEs, the role of which is probably limited to regulatory functions’ [13].

In this work, we revisited the role of *Alu* exonization in human proteome evolution and adopted a proteotranscriptomics approach [48] to systematically evaluate the evidence for *Alu* exon derived peptides in human proteins. We identified 262 putative coding *Alu* exons in Ensembl human transcripts, among which 85 exons had proteomic evidence in the PRIDE peptide database. Using multiple negative controls, we demonstrated that our proteomic identification of protein-coding *Alu* exons based on the PRIDE search had a low FDR. We also performed detailed analyses of RNA-seq and Ribo-seq data of human tissues and cell lines to provide more fine-grained information on the splicing and translational profiles of these protein-coding *Alu* exons. Using RNA-seq data of 19 human tissues, we identified 52 protein-coding *Alu* exons with high transcript inclusion levels and/or tissue-specific splicing profiles, significantly expanding the catalog of coding-region *Alu* exons with strong splicing activities in normal human tissues, which were considered rare in previous work [8, 9]. Collectively, our data challenge the conventional view on the proteomic impact of *Alu* exonization and suggest that an appreciable number of coding-region *Alu* exons are translated into stable protein products. Therefore, the contribution of *Alu* exons to the human proteome is significantly higher than previously suggested [13].

We studied a protein coding *Alu* exon in SUGT1, a gene encoding a cell cycle regulator [34]. The translation of this SUGT1 *Alu* exon is supported by multiple lines of evidence (Ribo-seq, PRIDE, PeptideAtlas) as well as previous literature [34–36]. Although the specific function of this SUGT1 *Alu* exon inclusion isoform remains to be elucidated, existing data suggest that the splicing of this *Alu* exon is under dynamic regulation in human cells. SUGT1 is reported to be a member of the pro-inflammatory complex ‘inflammasome’ and its protein level, especially the level of the *Alu* exon inclusion isoform (known as ‘SUGT1B’ in the literature), increases after heat shock [49]. SUGT1B also appears to be preferentially translocated to and accumulate in the nucleus under heat shock [50]. Additionally, there is anecdotal evidence showing SUGT1B is expressed at a much higher level than SUGT1A (that is, the *Alu* exon skipping isoform) in a human malignant glioblastoma cell line U-251 MG [51], as compared to the near or lower than 1:1 ratio in liver and tonsil tissues tested in the same experiment as well as various tissues and cell lines analyzed in other reports [34–36, 49]. Interestingly, our RT-PCR analyses indicate a significant gradient in the splicing levels of this SUGT1 *Alu* exon between human, chimpanzee, and rhesus macaque tissues, with the exon spliced at the highest levels in human tissues and completely skipped in rhesus macaque tissues (Fig. 3d). Therefore, this *Alu* exonization event has contributed to the acquisition and increased expression of a novel peptide segment during very recent human evolution. Of note, using a moderate-coverage six-tissue RNA-seq dataset of human, chimpanzee, and rhesus macaque [52], we identified two additional protein-coding *Alu* exons in SRP9 and ZNF468 (see Additional file 1: Table S1 for their annotations) with more than 15 % increase in splicing levels in at least one human tissue compared to the corresponding chimpanzee and rhesus tissue. Given the limited RNA-seq depth of this dataset, this list is expected to be quite incomplete.

## Conclusions

Our study has revealed a large list of *Alu* exons that may be translated and incorporated into primate-specific or even human-specific protein isoforms. These *Alu* exons are created in genes involved in a wide range of biological functions and molecular processes (Table 1). We selected an *Alu* exon in a transcriptome regulator ADARB1 and performed RNA-seq experiments to read out the activity of the *Alu* exon inclusion protein isoform on transcriptome-wide control of A-to-I RNA editing. For other protein-coding *Alu* exons identified in this work, future experiments tailored towards their genes’ specific cellular functions are needed to elucidate the evolutionary significance of the novel protein isoforms arising from *Alu* exonization.

## Materials and methods

### RNA-seq analysis of putative coding Alu exons

The locations of *Alu* elements in the human genome (hg19) were downloaded from the UCSC Genome Browser database [53]. The locations of internal cassette or constitutive exons were taken from Ensembl gene annotations (release 57) [54]. We defined an exon as *Alu*-derived if the *Alu* element covered at least 25 bp of the exon and more than 50 % of the total exon length.

We downloaded the Human Body Map 2.0 (HBM2.0) paired-end RNA-seq data from Gene Expression Omnibus (GEO) (accession number GSE30611). HBM2.0 RNA-seq data have a read length of 50 bp. They cover 16 tissues: adipose, adrenal, brain, breast, colon, heart, kidney, liver, lung, lymph node, ovary, prostate, skeletal muscle, testes, thyroid, and white blood cells. We also used paired-end RNA-seq data of three anatomical compartments of the human placenta (amnion, chorion, and decidua) generated in our previous work [55]. We used only 50 bp of each end for mapping and analysis based on the sequencing error profile. We used the same RNA-seq mapping method as previously described [55] to obtain the splice junction read counts and calculate each exon’s transcript inclusion level. We used MATS to identify differential alternative splicing events in pairwise tissue comparisons [24].

### Search of the PRIDE database

We searched the PRIDE (PRoteomics IDEntifications) database [25] for peptide evidence for putative coding *Alu* exons. We downloaded the peptide sequences in June 2014 with more than 1.5 million unique peptide sequences in PRIDE. Of these 1.5 million peptide sequences, approximately 900,000 were uniquely mappable to the coding regions of the human Ensembl transcripts (release 57). With these uniquely mappable peptide sequences, we examined the ORFs (open reading frames) containing putative coding *Alu* exons and identified 85 *Alu* exons with PRIDE peptide evidence.

### Ribo-seq analysis of putative coding Alu exons

Ribo-seq data and corresponding RNA-seq data of HeLa cells were downloaded from [29]. The sequencing reads are 36 bp in length. We used the first 30 bp of each read for mapping and analysis. Reads were mapped to all splice junctions in human genes (Ensembl genes, release 57). Each splice junction is 54 bp in length, containing the last 27 bp of the upstream exon and the first 27 bp of the downstream exon. We used Bowtie [56] to map reads, allowing up to two mismatches and also required that each read should be uniquely mapped. Exons’ transcript inclusion levels were estimated as described previously [55].

### Construction of ADARB1 isoform expression vectors

The ORF for the human ADARB1 short isoform (without the *Alu* exon) in the pCMV6-AC vector was purchased from OriGene, Inc. (catalog no. SC321955; reference transcript NM_001112). Mutageneses using the QuikChange method (Stratagene) were carried out to convert the ORF to encode the ADARB1 reference protein sequence NP_001103. Six nucleotides in pCMV6-AC encoding two additional amino acids at the C-terminus were removed. A minor allele SNP (rs199697177) ‘C’ (allele frequency <1 %) was mutated back to the major allele ‘T’ in the ADARB1 ORF. Then the *Alu* exon was inserted using the same mutagenesis method. Final pCMV6-ADARB1-short (without the *Alu* exon) and pCMV6-ADARB1-long (with the *Alu* exon) constructs were confirmed by sequencing.

### Ectopic expression of ADARB1 isoforms in HEK293 cells

HEK293 cell line was grown in DMEM with 10 % FBS. Cells were transiently transfected with Lipofectamine 2000 reagent according to the manufacturer’s protocol. Forty-eight hours after transfection, cells were collected for RNA extraction using TRIzol reagent (Life Technologies) and protein lysates with RIPA buffer. Transfection experiments were replicated in three different cell passages.

Total RNA samples were treated with DNaseI (Fermentas) and reverse transcribed using the High-Capacity cDNA RT kit (Applied Biosystems). Quantitative real-time PCR (qRT-PCR) was performed using Fast SYBR green Master Mix (Applied Biosystems). Total gene expression level and isoform-specific expression level of ADARB1 were measured using GAPDH as the reference gene. Relative expression level was measured by the comparative Ct (2^-ΔΔCt^) method [57]. Primers used are:

GAPDH_F: 5′-TGGTATCGTGGAAGGACTCA-3′, GAPDH_R: 5′-ACAGTCTTCTGGGTGGCAGT-3′, ADARB1_Gene_F: 5′-AGTCTCCGCCAGTCAAGAAA-3′, ADARB1_Gene_R: 5′-GTTGTCCAGATTGCGGTTTT-3′, ADARB1_short_F: 5′-AGGCTGAAGGAGAATGTCCA-3′, ADARB1_short_R: 5′-TGTCTATCTGCTGGTTCTTC-3′, ADARB1_long_F: 5′-CTCAACCTTCCAAGGAGCTG-3′, ADARB1_long_R: 5′-GTCCGTAGCTGTCCTCTTGC-3′.

Total protein extract in RIPA buffer was used to assay for protein level. ADARB1 (sc-10012, Santa Cruz Biotechnology) and ACTB (A5441, Sigma) antibodies were used for western blot following standard protocol. Signals were detected by ChemiDoc™ MP imaging system (Bio-Rad). RNA-seq libraries were prepared using the TruSeq Stranded mRNA Sample Prep Kit (Illumina) and sequenced on an Illumina HiSeq 2000 (100 cycles, paired-end). RNA-seq data were deposited in the Gene Expression Omnibus database (http://www.ncbi.nlm.nih.gov/geo/) under the accession number GSE65999.

### RNA-seq analysis of A-to-I editing

Strand-specific paired-end RNA-seq reads were first mapped to Ensembl transcripts and the unmapped reads were then mapped to the human genome (hg19) using Tophat (version 1.4.1) [58]. We used REDItools [59] to calculate the edited and unedited read counts on the corresponding strand of each known RNA editing site collected in the RADAR database [41]. We removed the editing sites that had less than five edited counts in all the nine RNA-seq libraries to avoid using unreliable editing sites or sites not edited in the samples of interest. The sites that had no read coverage (edited counts and unedited counts) for all three replicates of either empty vector (EV), ADARB1 long form, or ADARB1 short form transfected cells were also removed from further analyses because they were uninformative for comparisons. In the end, 7,618 known editing sites were used in the analyses.

To compare the global editing levels between the three sample groups, we merged the RNA-seq reads of three replicates in each group, and the editing levels of each editing site were calculated as the fraction of edited counts over the total counts in the merged data. To avoid using sites with unreliable editing levels due to low read coverage, we used only the editing sites that had total read coverage ≥50. Then we calculated the significance for the editing level differences between two groups using two-tailed Wilcoxon test.

### Total RNA preparation

Postmortem tissue samples of three adult chimpanzees and three adult rhesus macaques were generously provided by the Southwest National Primate Research Center (San Antonio, TX, USA). Total RNAs were extracted using TRIzol (Invitrogen, Carlsbad, CA, USA) according to the manufacturer’s instructions. Total RNAs from various human tissues were purchased from Clontech (Mountain View, CA, USA), BioChain (Newark, CA, USA), and Ambion (now part of Thermo Fisher, Grand Island, NY, USA), or prepared as previously described [55].

### Fluorescently labeled RT-PCR analysis of exon splicing

Single-pass cDNA was synthesized using the High-Capacity cDNA Reverse Transcription Kit (Applied Biosystems, Foster City, CA, USA) according to manufacturer's instructions. Two micrograms of total RNA were used for each 20 μL cDNA synthesis reaction. Fluorescently labeled RT-PCR was carried out as described previously [60]. PCR primer sequences are listed in Additional file 4: Table S4. Each gel picture shown was a representation of at least three replicates.

### Minigene analysis of SUGT1 protein-coding *Alu* exon splicing

*Alu* exon (Exon 6) of SUGT1 and its adjacent flanking intronic regions were amplified from the human, chimpanzee and rhesus genomic DNAs using KAPA HiFi HotStart ReadyMix PCR Kit (Kapa Biosystems, Inc., Wilmington, MA, USA). PCR products were subcloned into the NotI site of the pI-11-H3 minigene vector [61] (kindly provided by Dr. Russ P. Carstens, University of Pennsylvania, Philadelphia, PA, USA) using the In-Fusion Advantage PCR Cloning Kit (Clontech, Mountain View, CA, USA). Sequence swapping mutagenesis [38] was done using KAPA HiFi HotStart ReadyMix PCR Kit. All sequences and mutations were verified by DNA sequencing.

### *In vitro* minigene splicing reporter assay

HeLa cells and chimpanzee fibroblast cells (S008861, Coriell Institute, Camden, NJ, USA) were grown in DMEM (Invitrogen, Thermo Fisher, Grand Island, NY, USA) with 10 % FBS (Invitrogen). Cells were plated in 12-well plates and transfected using Lipofectamine LTX and Lipofectamine 2000 (Invitrogen), respectively, according to the manufacturer’s protocol. RNA was purified 16 h after transfection and reverse-transcribed into single-pass cDNA. Fluorescently labeled RT–PCR was performed as described above. The pI-11-H3 minigene-specific primer sequences were pI11-F: 5′-GCTGTCTGCGAGGTACCCTA-3′; pI11-R: 5′-CGTCGCCGTCCAGCTCGACCAGCGTTCGGAGGATGCATAGAG-3′.

### Ethics statement

This work used post-mortem tissues obtained from the Southwest National Primate Research Center. The primate tissue repository was approved by the Institutional Animal Care and Use Committee at the Southwest National Primate Research Center at the Texas Biomedical Research Institute (Animal Welfare Assurance Number A3082-01; IACUC protocol number 525 PT for chimpanzees, and 525 MM for rhesus macaques).

## Additional files

Additional file 1: Table S1. Fifty-two putative coding *Alu* exons with moderate-to-high levels of exon splicing or differential splicing activities among human tissues. (XLSX 16 kb) Additional file 2: Table S2. Eighty-five putative coding *Alu* exons with peptide evidence from the PRIDE database. (XLSX 18 kb) Additional file 3: Table S3. Seventy-six putative coding *Alu* exons with at least 10 reads mapped to one of the three splice junctions in both Ribo-seq and RNA-seq data from HeLa cells. (XLSX 18 kb) Additional file 4: Table S4. List of RT-PCR primers. (XLSX 10 kb) Additional file 5:
**Supplementary figures S1-5.** (DOCX 2892 kb)

## Abbreviations

cDNA: complementary DNA
CDS: coding DNA sequence
DJC: downstream junction read count
EST: expressed sequence tags
FDR: false discovery rate
GEO: Gene Expression Omnibus
mRNA: messenger RNA
NMD: nonsense-mediated decay
PDB: Protein Databank
PRIDE: PRoteomics IDEntifications Database
PSI (Ψ): percent-spliced-in
PTC: premature termination codon
qRT-PCR: quantitative real-time PCR
Ribo-seq: ribosome profiling
RNA-seq: RNA sequencing
SJC: skipping junction read count
TE: transposable element
TPR: tetratricopeptide repeat
UJC: upstream junction read count
UTR: untranslated region
