# Supplementary material for: The contribution of Alu exons to the human proteome
Source: Genome Biol. 2016 Jan 28;17:15. doi: 10.1186/s13059-016-0876-5 (PMC4731929; doi:10.1186/s13059-016-0876-5)

Additional file 5:

Supplementary Figures

# **Figure S1**:

Gel images for fluorescently labeled RT-PCR analyses of 20 putative coding *Alu* exons in different human tissues. Band sizes corresponding to the *Alu* exon inclusion or skipping isoforms are indicated on the right.


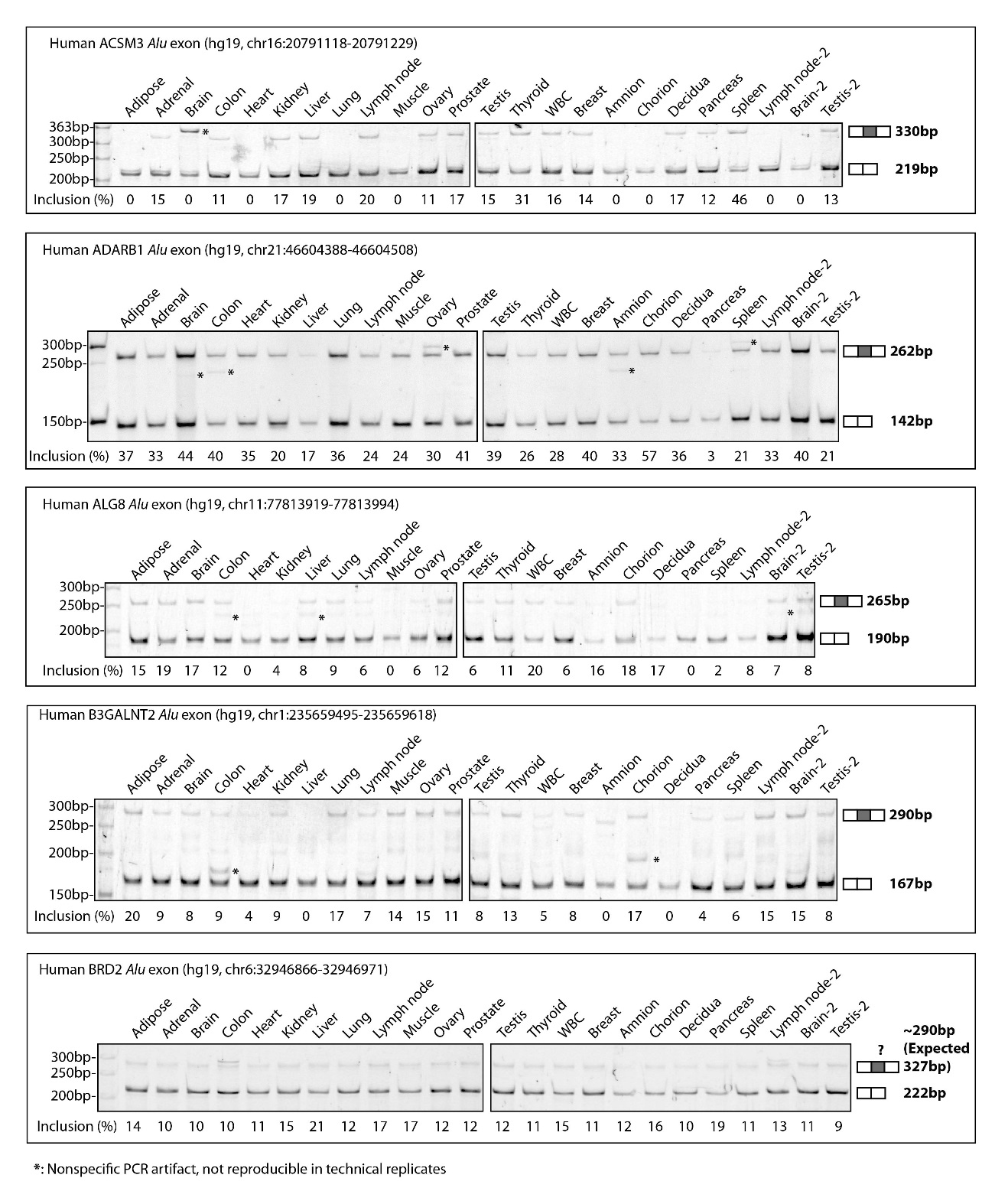


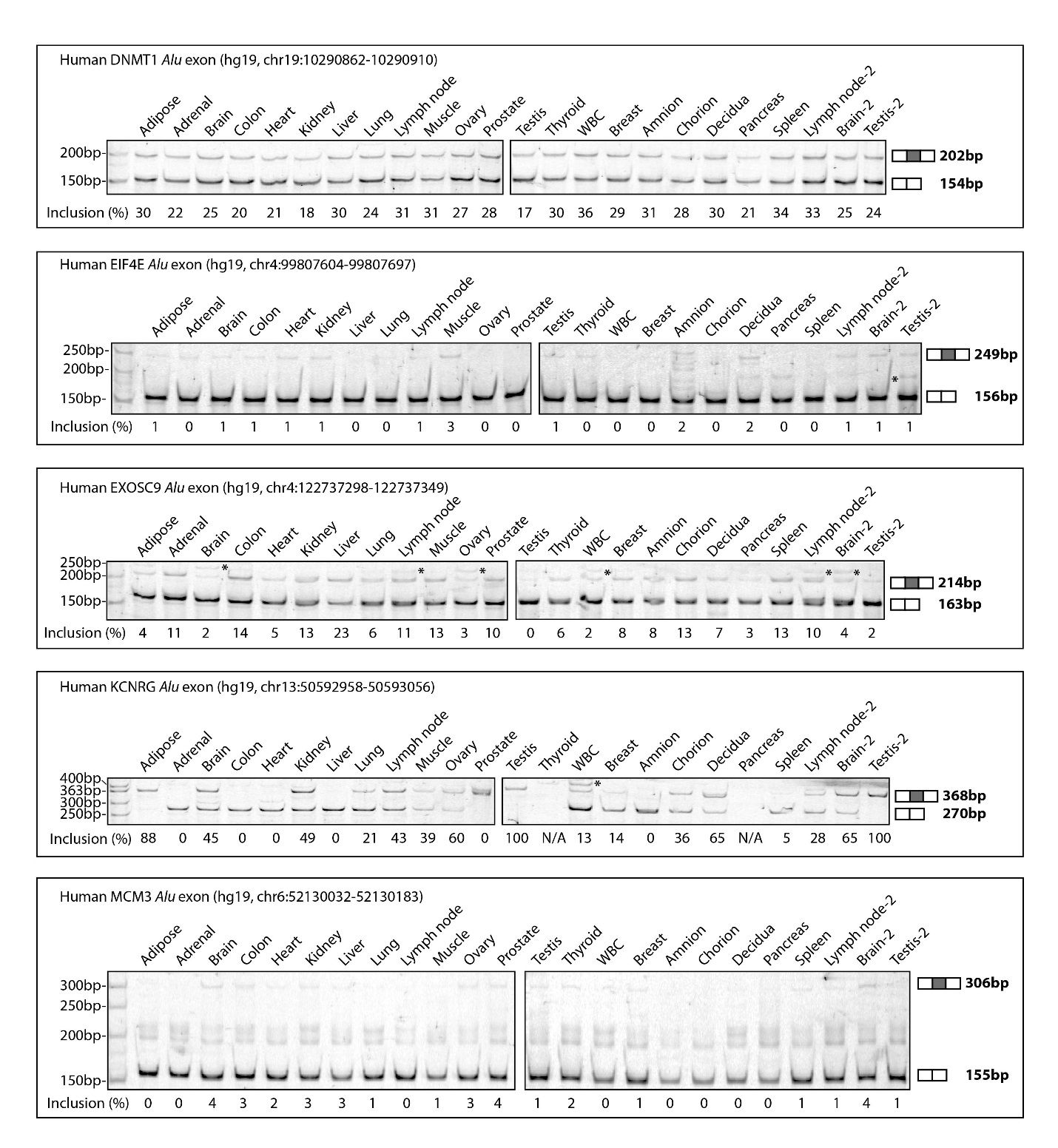


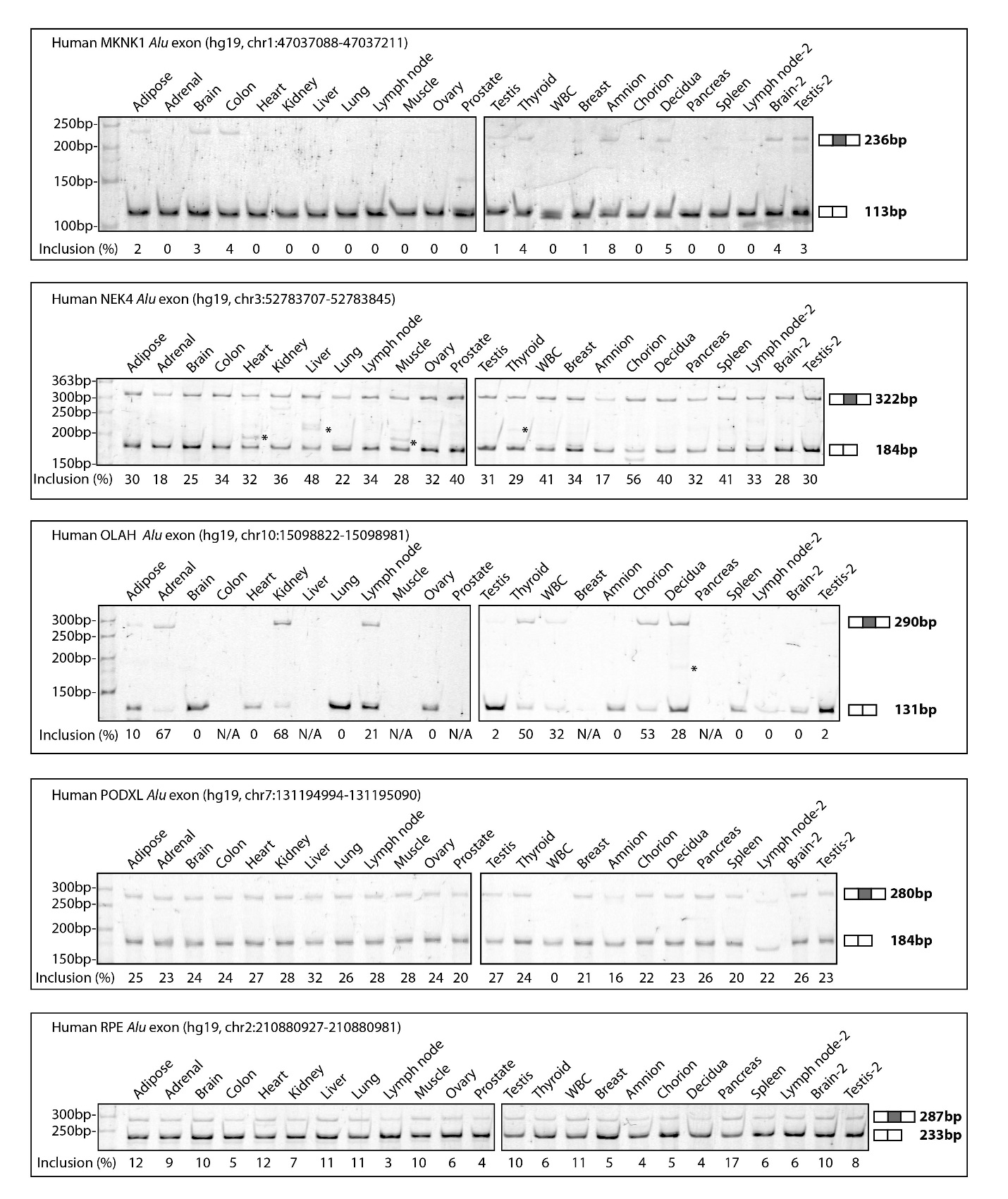


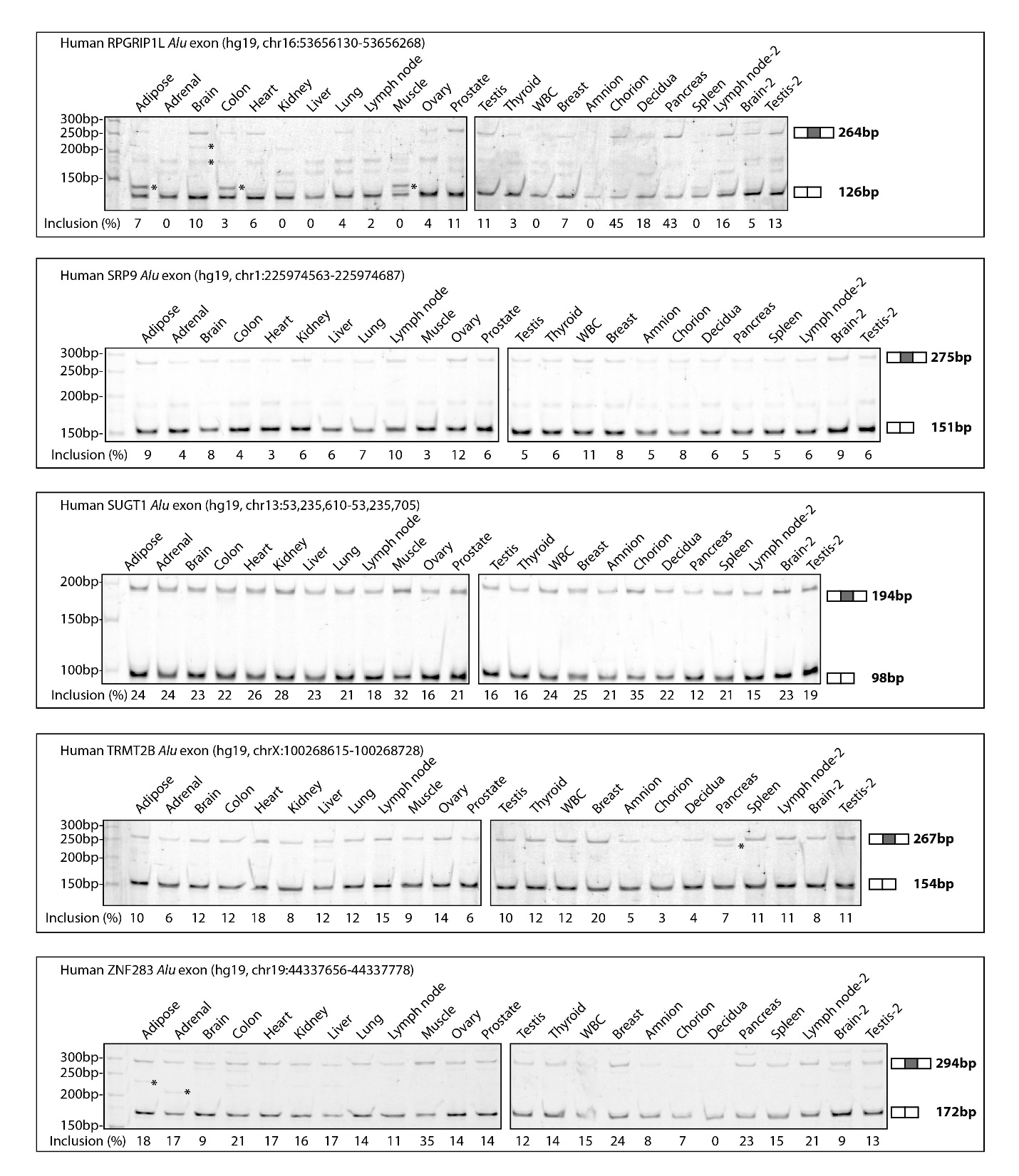


# **Figure S2:**

Gel images for fluorescently labeled RT-PCR analyses of the SUGT1 *Alu* exon in different tissues of human, chimpanzee (Chimp), and rhesus macaque (Rhesus). Band sizes corresponding to the *Alu* exon inclusion or skipping isoforms are indicated on the right.


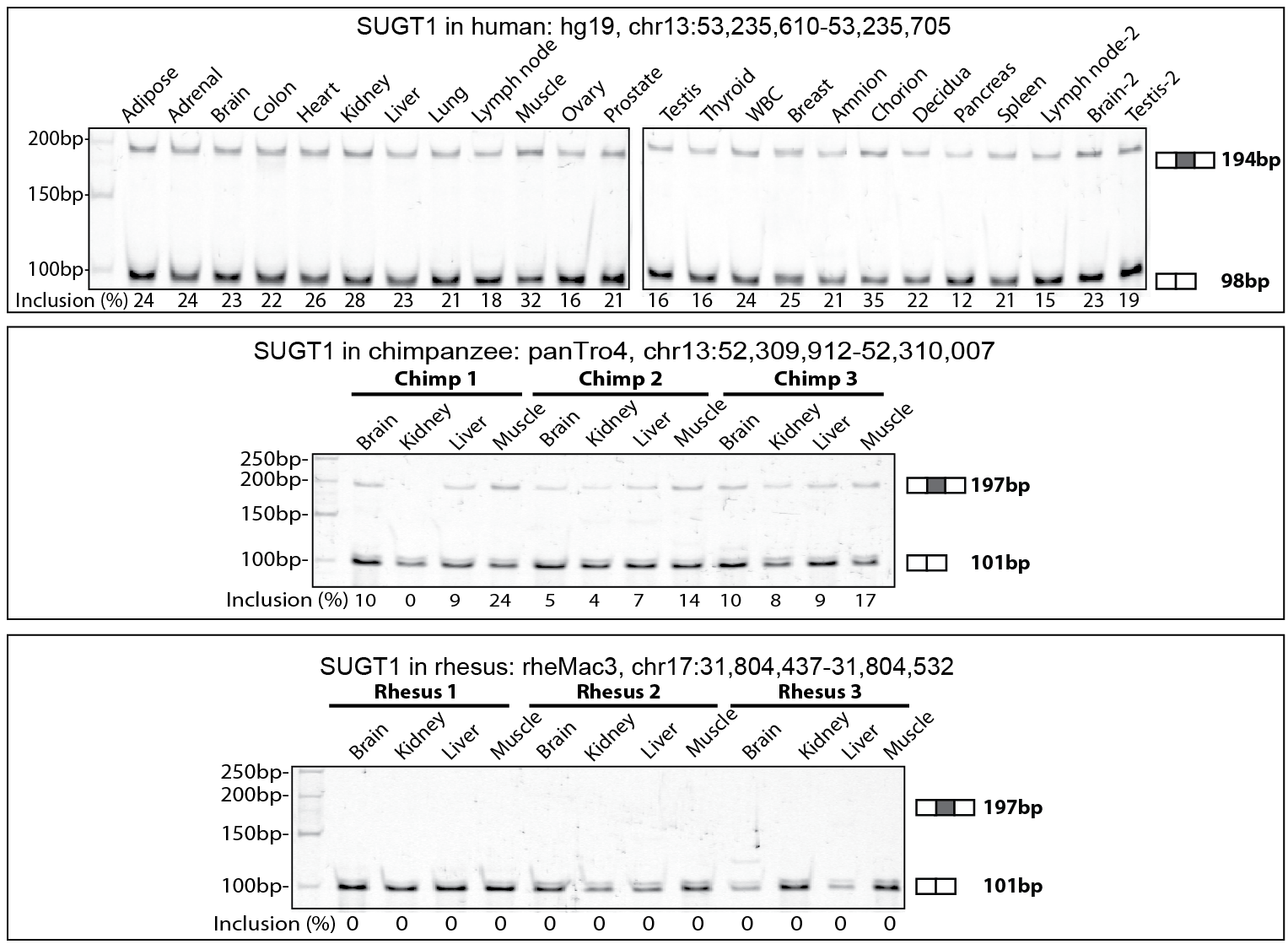


# Figure S3:

The multi-species alignment of genomic sequences surrounding the protein-coding *Alu* exon in SUGT1. Human (Hs), chimpanzee (Pt), and rhesus macaque (Rm) genomic sequences are aligned to the consensus sequences of AluSx1, 3, and 4. The genomic region corresponding to the *Alu* exon is indicated by the black box. The 3’ and 5’ splice sites are highlighted using white letters on a black background. The 9-nucleotide sequences used for calculating the MAXENT 5’ splice site scores are indicated in the red box. The G to A substitution at the +3 intronic position of the 5’ splice site is highlighted in red.


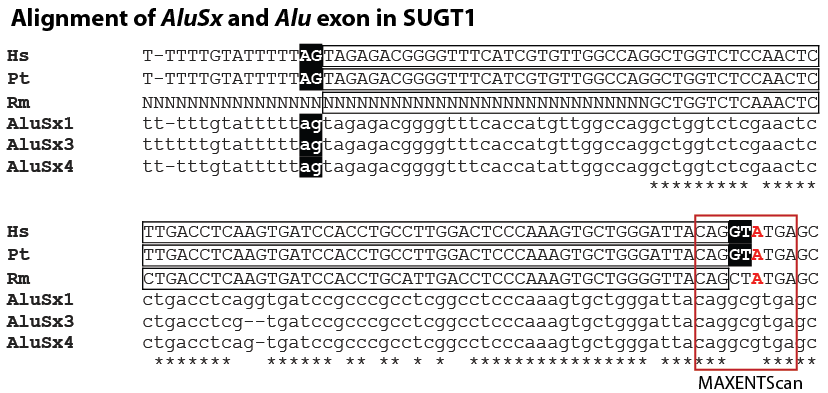


# **Figure S4**:

Gel images for fluorescently labeled RT-PCR analyses of the ADARB1 *Alu* exon in different tissues of human, chimpanzee (Chimp), and rhesus macaque (Rhesus). Band sizes corresponding to the *Alu* exon inclusion or skipping isoforms are indicated on the right.


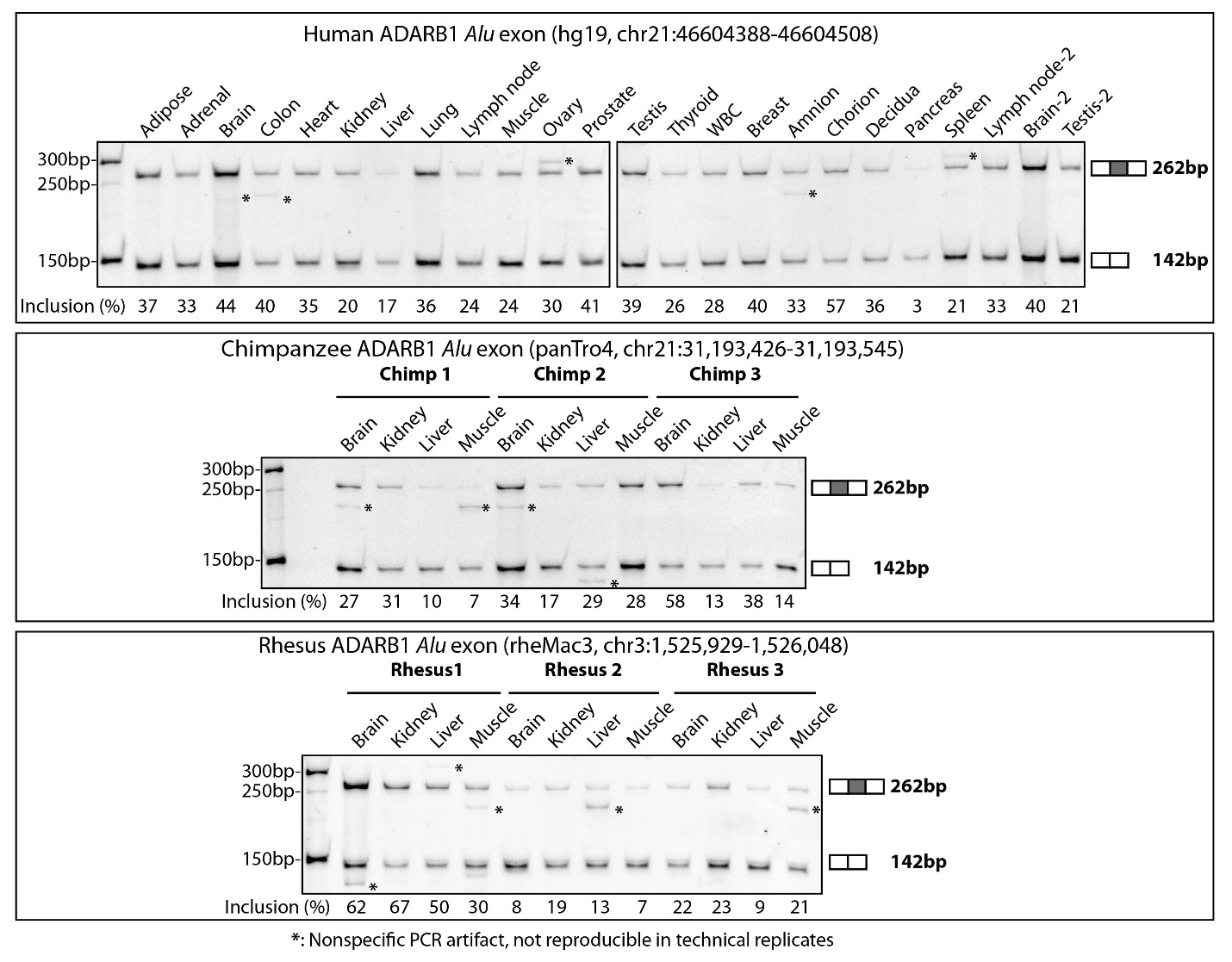


# Figure S5:

Ectopic expression of ADARB1 isoforms in HEK293 cells. HEK293 cells were transfected with empty vector (EV) control or plasmids with ADARB1 cDNA corresponding to the *Alu* exon skipping (Short) or inclusion (Long) isoform. Three replicated experiments were conducted. **a** qRT-PCR results for quantifying mRNA levels of ADARB1 isoforms. Three different sets of primers targeting the overall ADARB1 gene expression levels (that is, both isoforms), short isoform only, or long isoform only were used. The primer set targeting the overall ADARB1 gene expression levels (‘Gene Primer’) indicates comparable levels of total ADARB1 mRNA with the ectopic expression of either the exon skipping or inclusion isoform. Error bars show standard error of the mean from replicates. **b** Western blot analysis of ADARB1 protein levels.


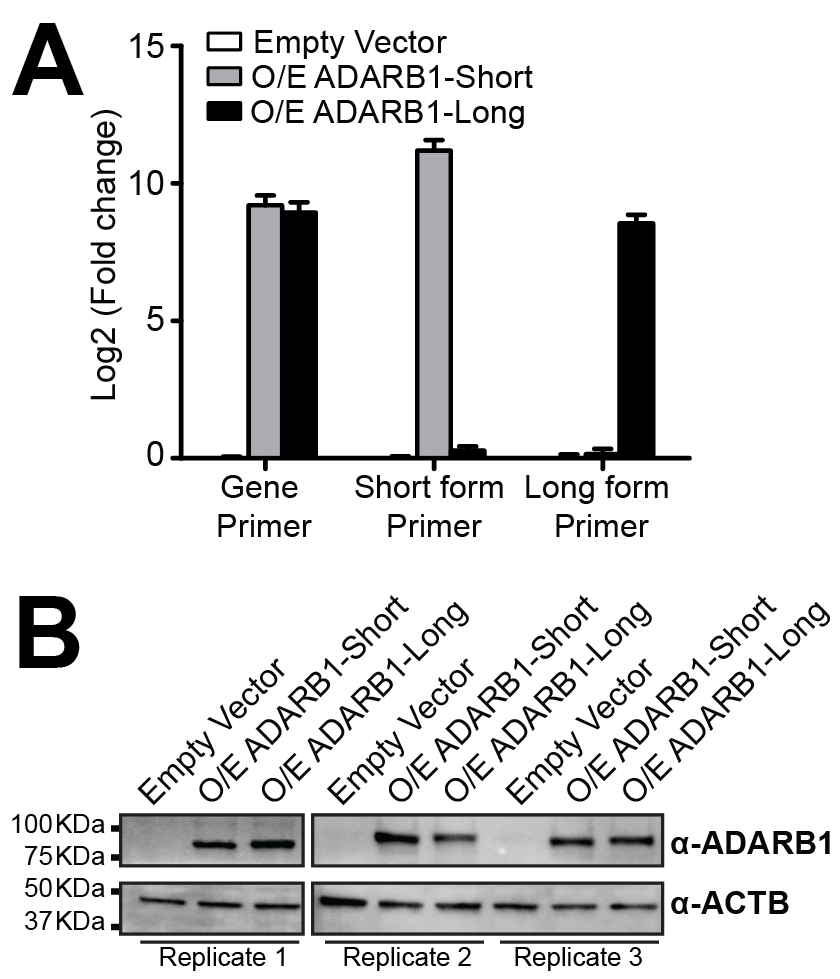

Supplement: Additional file 5: — Supplementary figures S1-5. (DOCX 2892 kb) [file 13059_2016_876_MOESM5_ESM.docx]
